# A Carboxyl-Functionalized Graphene Quantum Dot Coating for Catheters Effective Against Emerging Drug-Resistant *Candidozyma auris*

**DOI:** 10.3390/jof12030216

**Published:** 2026-03-17

**Authors:** Laure S. van Hofwegen, Muhammad Hassnain, Payal P. S. Balraadjsing, Karin van Dijk, Ferry Hagen, Sedat Nizamoglu, Sebastian A. J. Zaat

**Affiliations:** 1 Department of Medical Microbiology and Infection Prevention, Amsterdam Institute for Infection and Immunity, Amsterdam University Medical Centre, University of Amsterdam, 1105 AZ Amsterdam, The Netherlands; 2 Department of Biomedical Science and Engineering, Koç University, 34450 İstanbul, Türkiye; 3 Department of Medical Mycology, Westerdijk Fungal Biodiversity Institute, 3584 CT Utrecht, The Netherlands; 4 Department of Medical Microbiology, University Medical Center Utrecht, 3584 CX Utrecht, The Netherlands; 5 Institute for Biodiversity and Ecosystem Dynamics, University of Amsterdam, 1012 WP Amsterdam, The Netherlands; 6 Department of Electrical and Electronics Engineering, Koç University, 34450 İstanbul, Türkiye

**Keywords:** carboxyl-functionalized graphene quantum dots, photodynamic therapy, *Candidozyma auris*, *Candida albicans*, catheter coating, singlet oxygen, fungicidal activity

## Abstract

*Candidozyma auris* is an emerging opportunistic fungal pathogen that can cause serious catheter-related blood stream infections associated with high morbidity and mortality. The traditional antifungal treatment with polyenes, azoles or echinocandins is becoming less effective due to both intrinsic and developed resistance, complicating treatment. This study demonstrates the potent fungicidal activity of carboxyl-functionalized graphene quantum dots (cGQDs) against a panel of *C. auris* strains, spanning clades I to V, and a *Candida albicans* reference strain. Photoactivation of cGQDs in suspension with 435 nm blue light killed 99.9% of the fungi within 30 min even though the majority of test strains were resistant to at least one conventional antifungal. Moreover, cGQDs coated on flexible polydimethylsiloxane surfaces and commercial catheters via electrostatic layer-by-layer deposition with alternating positively charged polydiallyldimethylammonium polymer showed strong fungicidal activity against *C. auris* and *C. albicans*. These findings show that the cGQDs, both in suspension and in a thin film coating, have potential for future clinical development. In particular, their application to catheters may help prevent *Candidozyma* and *Candida* catheter-related infections.

## 1. Introduction

Catheter-related blood stream infections (CRBSIs) are one of the most common hospital-acquired infections with high mortality rates of 15–20% [1,2]. In 2021, the European Centre for Disease Prevention and Control reported that 38.3% of blood stream infections in intensive care units are related to catheters [3]. CRBSIs specifically associated with central venous catheters were caused by the fungal *Candida* species in 29.3% of cases in US acute-care hospitals. Other common causative pathogens found were coagulase-negative staphylococci (13.3%) and *Enterococcus faecium* (8.1%) [4]. *Candida* species include *Candida albicans* (*C. albicans*) but also *Candidozyma auris* (*C. auris*), previously known as *Candida auris* [5]. *C. auris* is an emerging pathogenic yeast with high rates of intrinsic resistance to conventional antifungals [6]. Besides the difficulty of treating CRBSIs due to antifungal resistance, these yeasts also have a strong ability to colonize the skin and form persistent biofilms on a variety of surfaces, including surfaces in hospital environments [7,8]. The limited efficacy of antiseptics may have actually caused the selection of *C. auris* as a nosocomial pathogen, leading to the spread between patients and resulting in healthcare-associated outbreaks [8]. In 2022, *C. auris* was categorized in the critical priority group of fungal pathogens by the World Health Organization as a major worldwide threat to public health [9]. Based on its geographic and genetic origins, *C. auris* strains are classified into six clades: Clade I from South Asia, Clade II from East Asia, Clade III from South Africa, Clade IV from South America, Clade V from Iran, and Clade VI from Singapore [10,11]. In particular, isolates from Clades I, III and IV have been reported to have high resistance to at least one of the antifungal classes [12,13,14,15].

The high morbidity and mortality rates, especially among immunocompromised patients, underline the need for novel and more effective prevention and treatment strategies than those currently available [16]. A variety of approaches such as novel antifungals with new mechanisms of action, combination therapy or drug repurposing have been applied [17]. However, the risk of developing resistance for these agents is high for single-target antifungals and they often do not have broad-spectrum activity or rely on synergy with conventional antifungals [17]. Recently, antimicrobial photodynamic therapy (aPDT) emerged as a promising strategy against fungal species [18]. aPDT depends on the interaction between light and a photosensitizer in the presence of oxygen to produce reactive oxygen species (ROS) [18]. However, the currently available photosensitizers have low biocompatibility and require high-intensity light which causes skin sensitization [19].

Graphene quantum dots (GQDs) are a promising class of nanomaterial photosensitizers owing to their high biocompatibility [20] and requirement for only moderate- or low-intensity light for their activation. GQDs consist of a single layer of carbon atoms arranged in a honeycomb-like structure which can be functionalized with different chemical groups to maximize antimicrobial activity. Recently, we developed carboxyl-functionalized GQDs (cGQDs) with strong activity against multi-drug-resistant *Staphylococcus aureus* and *Escherichia coli* [21]. Photo-activation with low-intensity blue light generated exceptionally high levels of singlet oxygen, which effectively killed both bacterial species. Although cGQDs are very effective against bacterial species, they have not been tested for antifungal activity against *C. albicans* or drug-resistant *C. auris* or applied to relevant clinical material such as catheters.

In the present study, we investigated the effectiveness of cGQD thin films to eliminate *C. auris* and *C. albicans* adhering to catheters. We first evaluated the antifungal activity of cGQDs in a colloidal state against *C. albicans* and a panel of *C. auris* strains spanning clades I to V, and subsequently incorporated the cGQDs into a thin film on glass or PDMS. Finally, we applied the cGQD thin film to peritoneal/cardiac catheters and assessed the fungicidal activity to confirm potential clinical application for the prevention of *C. albicans* or *C. auris* infections.

## 2. Materials and Methods

### 2.1. Fungi

Strains of *C. auris* used in this study were 111, 2MG-A0102-44, 2MG-A0102-51, 2MG-A0202-80, 2MG-A0203-98, 2MG-A0203-50, 2MG-A0203-512MG-A0203-99, 2MG-A0203-100, 2MG-A1002-43 and 2MG-A1703-88. The antifungal sensitive reference *C. albicans* SC5314 strain was described previously [22]. Susceptibility to conventional antifungals fluconazole, voriconazole, itraconazole, isavuconazole, posaconazole, amphotericin B and micafungin (Sigma-Aldrich, St. Louis, MO, USA) was determined by the broth microdilution method, according to European Committee on Antimicrobial Susceptibility Testing (EUCAST) guidelines [23]. The minimum inhibitory concentration (MIC) was determined as the lowest concentration that inhibited visual growth of yeast. Yeasts were stored in tryptic soy broth (TSB, BD Difco, Franklin Lakes, NJ, USA) with 20% (*v*/*v*) glycerol at −80 °C.

### 2.2. cGQD Synthesis

The cGQDs were synthesized as described previously [21]. Briefly, pyrene (Sigm-Aldrich) was converted into 1,3,6 trinitropyrene. After cooling, the acidic contents were removed by diluting with deionized (DI) water and filtering with 0.22 μm filter paper (ISOLAB Laborgäte GmbH, Eschau, Germany). The 1,3,6 trinitropyrene residue on the filter paper was dried to a powder in a vacuum chamber. For carboxyl group functionalization on the edges of the GQDs, the dried 1,3,6 trinitropyrene was dispersed in aqueous solution of sodium bicarbonate (NaHCO_3_, Sigma-Aldrich) and sonicated for 1 h until the solute was homogenous in the solvent. The suspension was autoclaved, put into a furnace and heated at 200 °C for 10 h. After cooling down, the solution was filtered with 0.22 μm filter paper to remove large particles and other residual elements, and the filtrate dialyzed for 3 days with 3.5 K MWCO SnakeSkin Dialysis Tubing (Thermo Fisher Scientific, Waltham, MA, USA). The final solution was stored for further use.

### 2.3. Fabrication of cGQD Thin Film on Glass

The cGQD thin film on glass was fabricated as described previously [21]. Briefly, indium tin oxide (ITO, Ossila Ltd., Sheffield, UK) substrates were cleaned and coated with TiO_2_ using a spin coater (POLOS GmbH, Karlsruhe, Germany). The cGQD solution was dip coated onto the ITO/TiO_2_ surface, followed by a DI water rinse. Subsequently, the polydiallyldimethylammonium chloride (PDDA, Sigma-Aldrich) solution, which was prepared separately, was dip-coated onto the surface, followed by a DI water rinse. This deposition cycle was repeated four times and finished with a final layer of cGQDs to achieve five layers of cGQDs with four separation layers of PDDA in between. Finally, samples were dried and stored at room temperature for further use.

### 2.4. Fabrication of cGQD Thin Film on PDMS

PDMS Sylgard 184 silicone elastomer kit (Dow Corning Corporation, Midland, MI, USA) consists of pre-polymer and cross-linker components to produce heat-curable PDMS. The pre-polymer and cross-linker were poured into a Petri dish at a weight ratio of 10:1. The mixture was stirred manually for 5 min, left to rest for 5 min, and placed into a vacuum desiccator (ISOLAB Laborgäte GmbH) for 10 min to degas and remove bubbles. The composite was cured overnight in an oven at 60 °C. The PDMS sheets were cut into smaller pieces and processed with oxygen plasma treatment (50 W, 30 s, room temperature) to increase hydrophilicity and improve the coating of water-based solutions. The plasma treated PDMS samples were spin-coated with TiO_2_ and the coating was annealed at 120 °C for 30 min. The PDMS/TiO_2_ samples were again subjected to plasma treatment, followed by layer-by-layer dip-coating with 5 layers of cGQDs, with layers of PDDA polymer in between the GQD layers. Finally, the GQD-PDMS thin films were rinsed with water, dried in an oven at 60 °C and stored at room temperature in cell culture plates for further use.

### 2.5. Fabrication of cGQD Thin Film on Peritoneal/Cardiac Catheters

Commercial peritoneal/cardiac catheters (MEDTRONIC, 43103, WestCMR, Clearwater, FL, USA) were used as substrates for the fabrication of cGQD thin films. Prior to coating, catheters were rinsed with DI water and ethanol, dried, and surface-activated by UV–ozone treatment (Ossila Ltd.) for 10 min. For TiO_2_ dip coating, a TiO_2_ paste (25 mg mL^−1^, Greatcell Solar Materials Pty Ltd., Queanbeyan, NSW, Australia) was prepared by diluting TiO_2_ paste in absolute ethanol, and the activated catheters were immersed in the dispersion for 20 min, rinsed with absolute ethanol to remove excess material, and dried vertically in an oven at 80 °C. The TiO_2_-coated catheters were then re-treated with UV–ozone for 10 min, followed by layer-by-layer dip coating to assemble alternating cGQD and PDDA layers; a total of five cGQD layers and four PDDA separation layers were deposited. After the final round of dip coating, the catheters were rinsed three times with fresh DI water to remove loosely bound species. Finally, the coated catheters were dried vertically at 80 °C and stored for further characterization and antifungal experiments.

### 2.6. Characterization of Colloidal cGQDs and Thin Films

Transmission electron microscope (TEM) and high-resolution transmission electron microscope (HRTEM) images were acquired using a HITACHI HF5000 (Hitachi High-Tech Corporation, Tokyo, Japan) operating at 200 kV. For cross-sectional imaging of the PDMS–cGQD film and catheter–cGQD film, the samples were treated with liquid nitrogen for 30 s and fractured. For improved SEM imaging, a thin gold coating was applied by sputtering, and images were captured using a ZEISS Ultra Plus FE-SEM (Carl Zeiss AG, Oberkochen, Germany). Absorbance profiles of colloidal cGQDs and PDMS-cGQD film were recorded using a Synergy H1 microplate reader (BioTek, Winooski, VT, USA). For the stability analysis of the film, the initial absorbance was recorded using 500 μL of DI water. A PDMS–cGQD film (14 mm^2^) was immersed in a well of a 24-well plate. At each subsequent time point, 500 μL of the eluate was transferred to a new well, and its absorbance was measured. The same 500 μL of eluate was then returned (each time) to the original well containing the PDMS–cGQD film. Singlet oxygen generation by the PDMS–cGQD film was evaluated using 9,10-anthracenediyl-bis(methylene)dimalonic acid (ABDA; Sigma-Aldrich) as a singlet oxygen probe. In a 24-well plate, PDMS–cGQD or PDMS films (14 mm^2^) were placed in 1 mL of PBS containing ABDA (final concentration 25 μM). The plates were either kept in the dark or illuminated with a 455 nm light source (Thorlabs, Newton, NJ, USA) controlled by a Thorlabs LED driver for high-power mounted LEDs. At defined time intervals, absorbance spectra from 300 nm to 550 nm were recorded using a Synergy H1 microplate reader (BioTek, Winooski, VT, USA). For singlet oxygen measurements under multiple activation cycles, duplicate catheter–cGQD films (1 cm segments) were immersed in 500 μL ABDA solution (25 μM in PBS) in a 24-well plate. The samples were illuminated for 30 min (round 1), then rinsed with DI water, transferred to freshly prepared ABDA solution, and illuminated again for 30 min (round 2). The same procedure was repeated for a third illumination cycle (round 3). During each cycle, ABDA absorbance at 380 nm was recorded at defined time intervals using a Synergy H1 microplate reader (BioTek, Winooski, VT, USA). Singlet oxygen proportionally reduced this ABDA absorbance signal.

### 2.7. Minimal Fungicidal Concentration (MFC) Assay

Yeasts were cultured to mid-logarithmic growth phase at 37 °C with shaking at 120 rpm and diluted with PBS to 1 × 10^7^ CFU/mL, based on the optical density (OD) at 620 nm. The exact number of CFUs added to the experiment (inoculum) was determined by quantitative plating of quadruplicate 10 μL droplets of 10-fold serial dilutions on blood agar plates and incubating overnight at 37 °C for colony counting the next day. Yeast suspension (final concentration of 1 × 10^6^ CFU/mL) was added as a 10 μL volume to 90 μL of colloidal cGQDs (final concentration range of 3.1–25 μg/mL) in polystyrene 48-well flat bottom plates (VWR, Radnor, PA, USA). Growth control was yeast in PBS without cGQDs. The plate was kept in the dark or illuminated with 435 nm blue light from above at 3 cm distance from the sample under stationary conditions for 30 min. After incubation, 10 μL samples were taken from each well for 10-fold serial dilutions, which were plated on blood agar plates, incubated overnight at 37 °C and colonies were counted the next day. The minimal fungicidal concentration (MFC) was defined as the concentration of cGQDs that killed at least 99.9% (3-log) of the inoculum. All mentions of log refer to log10 values. The detection limit was 20 CFUs.

### 2.8. Surface Fungicidal Activity Assay for cGQD Thin Films

The Japanese Industrial Standard assay Z 2801:2000 [24] was used with modifications to assess surface fungicidal activity of cGQD-coated glass materials. In short, yeasts were cultured to mid-logarithmic phase at 37 °C with shaking at 120 rpm and diluted with PBS to 1 × 10^6^ CFUs, based on the OD620 nm. The exact inoculum added to the experiment was assessed by quantitative culture as described above. The cGQD material was placed in a well plate and the fungal suspension, in a volume of 50 μL containing 4.5 × 10^4^ CFUs (for glass slides of 3 cm^2^) or 25 μL containing 2.3 × 10^4^ CFUs (for PDMS samples of 1.5 cm^2^), was added and spread out to cover at least 90% of the material. The number of yeast cells added to the material was calculated based on the JIS Z 2801:2000 protocol, where 1.5 × 10^4^ CFUs are applied per cm^2^. For the catheter samples, the catheter was cut into pieces of 1 cm and then longitudinally cut in half to place them flat. Three droplets of 1 μL containing 1 × 10^4^ CFUs in total were placed on the catheter surface. Materials were either kept in the dark for 30 min or illuminated from above at 3 cm distance from the cGQD thin film surface with 435 nm blue light (5 mW/cm^2^, LumiSource, PCI Biotech, Oslo, Norway). Subsequently, PBS was added to each sample until fully submerged and then sonicated for 5 min at 35 kHz in a sonicator water bath (Transsonic 460, Elma, Singen, Germany) to detach yeast cells. Sonicate fluid was collected in Eppendorf tubes and 100 μL of the undiluted and serial ten-fold dilutions were plated on blood agar. Blood agar plates were incubated overnight at 37 °C and numbers of CFUs were counted the next day. The limit of detection was 20 CFUs for the glass and PDMS samples and 10 CFUs for the catheter samples. In addition, to assess whether any live yeast remained attached to the glass surface after sonication, the material was incubated overnight in TSB on a shaker at 120 rpm at 37 °C. Visual growth, observed as turbidity the next day, indicated residual live attached cells after the treatment and sonication procedure.

### 2.9. Statistics

All statistical analyses were performed in Graphpad Prism (v10.6.0). Comparisons were made using two-way ANOVA with uncorrected Fisher’s Least Significant Difference. All *p*-values of ≤0.05 were considered significant.

## 3. Results

### 3.1. Colloidal cGQDs and Their Antifungal Mechanism

**Figure 1:**
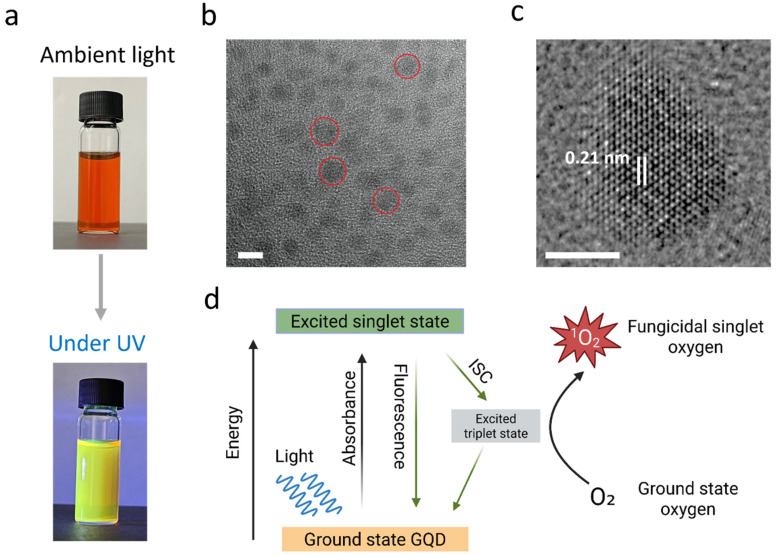
Characterization of cGQDs and their antifungal mechanism. (**a**) Photograph of cGQDs dispersed in water under ambient light and under ultraviolet (UV) light exposure. (**b**) TEM image of cGQDs, scale bar: 5 nm. Representative cGQDs are highlighted with red circles. (**c**) HRTEM image with interplanar spacing, scale bar: 5 nm. (**d**) Schematic illustration of singlet oxygen formation and fungicidal mechanism.

We synthesized cGQDs via hydrothermal synthesis using pyrene as a carbon source. The resulting water-dispersed cGQDs formed a brownish suspension under ambient light and exhibited green photoluminescence under UV light (Figure 1a), with a homogeneous size distribution and an average nanoparticle diameter of 5.1 nm (Figure 1b). High-resolution transmission electron microscopy (HRTEM) revealed well-defined hexagonal benzene rings fused into an extended honeycomb lattice, forming nanoscale graphene quantum dots with an interplanar spacing of 0.21 nm, consistent with the (100) lattice plane (Figure 1c). Upon light exposure, ground-state electrons of the cGQDs absorb photons with energy exceeding the bandgap, transition to a short-lived excited state, and then return to the ground state releasing excess energy as photons (Figure 1d). Due to the –COOH functionalization of GQDs, the majority of the excited electrons undergo intersystem crossing to form an intermediate excited triplet state [21], which transfers energy to surrounding oxygen molecules, forming singlet oxygen (^1^O_2_).

### 3.2. The Fungicidal Activity of Colloidal cGQDs

**Figure 2:**
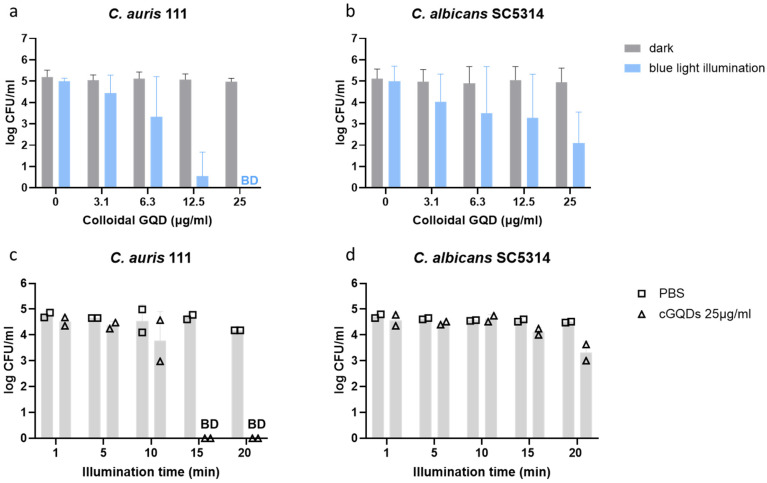
Fungicidal activity and minimal necessary illumination time of colloidal cGQDs. Fungicidal activity of cGQD against (**a**) *C. auris* and (**b**) *C. albicans* in the dark or after 30 min of blue-light illumination. Bars represent mean values of four experiments with duplicate measurements. Error bars represent standard deviation. Illumination time series of cGQDs at 25 μg/mL against (**c**) *C. auris* and (**d**) *C. albicans*. PBS was used as a negative control without cGQDs. Bars represent mean values of one experiment with duplicate measurements. Survival of yeasts is shown in log CFU/mL. Detection limit is 20 CFUs. BD; below detection limit.

To assess the fungicidal activity of colloidal cGQDs, we performed the minimum fungicidal concentration (MFC) assay with *C. auris* and *C. albicans*. After 30 min of illumination, the colloidal cGQDs killed at least 99.9% of the inoculum at concentrations of 12.5–25 μg/mL for *C. auris* and 25 μg/mL for *C. albicans* (Figure 2a,b). To study the minimal necessary illumination time, we subsequently performed the MFC assay with different illumination times of 1, 5, 10, 15 and 20 min at a concentration of 25 μg/mL cGQDs. The entire *C. auris* inoculum was killed after 15 min of illumination (Figure 2c). However, illuminating for 20 min was not enough to reach a 99.9% reduction in *C. albicans* (Figure 2d). Taken together, these data show strong fungicidal activity against both *C. auris* and *C. albicans* upon 30 min of illumination, where illumination time could be reduced to 15 min for treating *C. auris*.

### 3.3. Susceptibility to cGQDs of C. auris Strains of Different Clades

To assess susceptibility to colloidal cGQDs across the genetic clades of *C. auris*, we analyzed a panel of ten *C. auris* strains, comprising three clade I strains and two strains from each of clades II–V (except clade VI, which is not available to the research community). We first characterized the strains for their susceptibility to conventional antifungals (Table 1). Almost all *C. auris* strains had high MIC values for fluconazole, almost all clade I, III, IV and V strains were resistant to micafungin and one clade I strain was resistant to amphotericin B. Susceptibility to colloidal cGQDs was assessed using the MFC assay with 30 min of illumination and almost all strains showed 99.9% reduction in CFUs at a cGQD concentration of 12.5 μg/mL (Table 2). Only the two strains of clade III showed 99.9% reduction at 25 μg/mL. However, we observed a variation range between 12.5 and 25 μg/mL in repeated experiments with *C. auris* 111 and, therefore, we conclude from these data that there is essentially no difference in susceptibility to colloidal cGQDs between the strains of the *C. auris* clades. All strains, independent of their conventional antifungal resistance profile, were effectively killed by cGQDs.

### 3.4. The Fungicidal Activity of cGQD Thin Films on Glass

**Figure 3:**
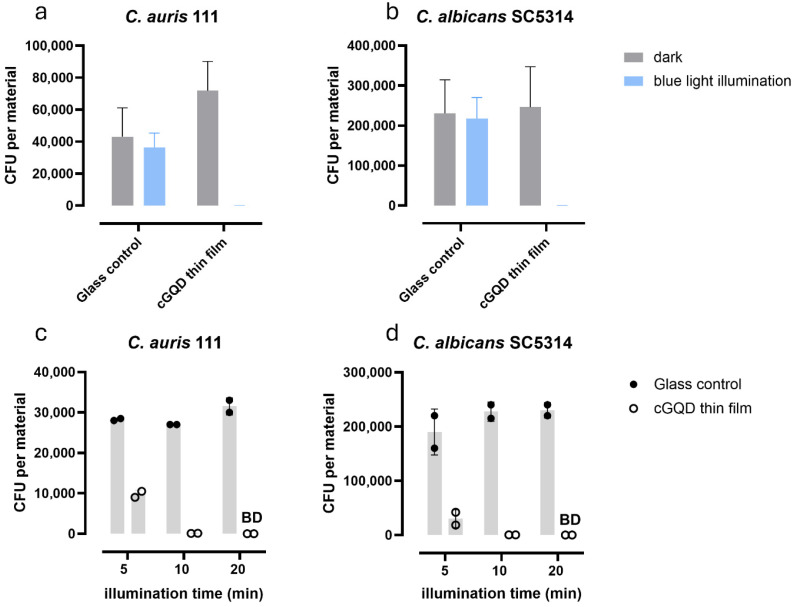
Fungicidal activity and minimal necessary illumination time of cGQD thin film on glass. Fungicidal activity in the dark or after 30 min of blue-light illumination against (**a**) *C. auris* and (**b**) *C. albicans*. Bars represent mean values of two experiments with duplicate measurements. Error bars represent standard deviation. Illumination time-series of cGQD thin film against (**c**) *C. auris* and (**d**) *C. albicans*. Bars represent mean values of one experiment with duplicate measurements. Survival of yeasts is shown in CFUs per material. Detection limit is 20 CFUs. Note slight differences in scale between panels. BD; below detection limit.

To study the capacity of the cGQDs to prevent survival on surfaces, we incorporated the cGQDs into a cGQD thin film on glass. The thin film was composed of five layers of GQDs with intermittent layers of PDDA polymer. The fungicidal activity was assessed using the Japanese Industrial Standard (JIS) assay. After 30 min of illumination, no remaining CFUs were found when yeasts were applied on the cGQD thin film, neither of *C. auris* nor of *C. albicans* (Figure 3a,b). The glass control and the dark condition showed no significant decrease in CFUs for either of the species. To study the minimally necessary illumination time, we performed the JIS assay with different illumination times of 5, 10 and 20 min. At least 99.9% reduction in CFU numbers was found after as little as 10 min of illumination for both *C. auris* and *C. albicans* (Figure 3c,d). These data show strong surface fungicidal activity of the cGQD thin films against both *C. auris* and *C. albicans*, with a minimum illumination time of 10 min.

### 3.5. Characterization of cGQD Thin Film on PDMS

**Figure 4:**
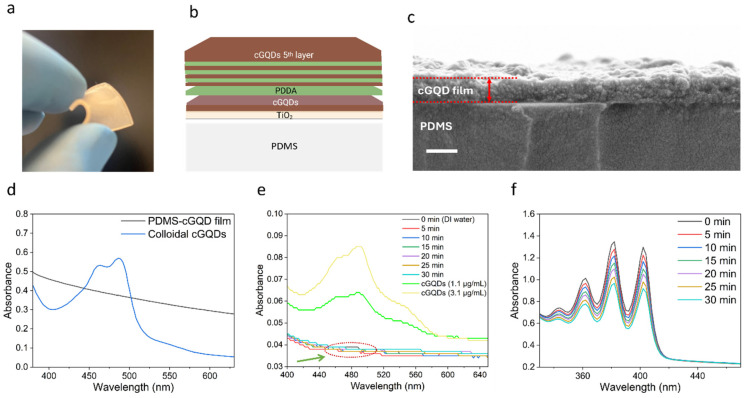
Characterization of cGQD thin film on PDMS. (**a**) Flexible cGQD thin film on PDMS. (**b**) Layer-by-layer deposition of cGQDs and PDDA by dip-coating. (**c**) SEM cross-sectional view of cGQD thin film, size bar: 400 nm. (**d**) Absorption profile of PDMS-cGQD thin film compared to 25 μg/mL colloidal cGQDs. (**e**) UV–vis release analysis of cGQDs from PDMS–cGQD thin films at different time points (0–30 min). The constant absorbance (arrow) indicates high coating stability and negligible cGQD release. Spectra of free cGQD suspensions (1.1 and 3.1 μg/mL) are included as controls. (**f**) Absorption spectra of ABDA singlet oxygen probe for PDMS-cGQD thin film measured at 5 min intervals over 30 min of blue-light illumination, showing reduction in the absorbance over time.

**Figure 5:**
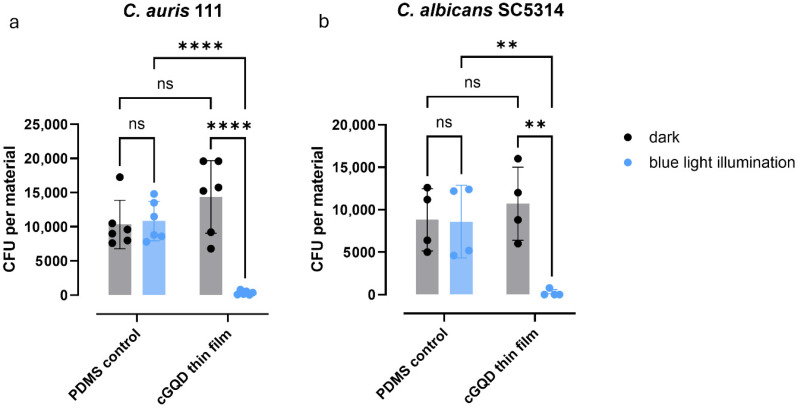
Fungicidal activity of PDMS-cGQD thin films. Fungicidal activity of PDMS-cGQD thin film in the dark or with 30 min of blue-light illumination against (**a**) *C. auris* and (**b**) *C. albicans*. Bars represent mean values of two or three experiments with duplicate measurements. Error bars represent standard deviation. Detection limit is 20 CFUs. ns; not significant, **; *p* ≤ 0.01, ****; *p* ≤ 0.0001.

To assess the applicability of cGQDs to a surface relevant for future medical application (e.g., catheters), the cGQD thin film was applied on PDMS (Figure 4) and subsequently tested for fungicidal activity (Figure 5). The cGQDs incorporated on a flexible and stretchable PDMS surface through the layer-by-layer deposition method is presented in Figure 4a. We first deposited a titanium oxide (TiO_2_) base layer followed by dip-coatings of negatively charged cGQDs and cationic polymer PDDA to create a stable and sufficiently thick film for antifungal activity (Figure 4b). A cross-sectional view of the PDMS-cGQD film showed a uniform thickness of 316 nm, including the TiO_2_ base layers, PDDA intermediate layers, and cGQD photoactive layers (Figure 4c). The combined cGQD and PDDA layers appeared as a single continuous layer because the individual layers could not be distinguished due to their similar carbon-based elemental composition. cGQDs absorb light in the visible light wavelength range with a pronounced absorption profile in the blue spectral zone. When cGQDs were incorporated into the multilayered film, the absorption peaks were diminished but the film still showed an absorbance at 445 nm, equal to that of colloidal cGQDs (25 μg/mL) (Figure 4d). The PDMS–cGQD film demonstrated excellent stability under aqueous conditions, with no detectable leaching of its deposited components (Figure 4e). We analyzed the singlet oxygen generation ability of the PDMS–cGQD film under blue light using bleaching of the ABDA probe. The spectra at increasing time points showed progressive bleaching of ABDA in the presence of the PDMS–cGQD film, indicating the continued production of singlet oxygen (Figure 4f). No singlet oxygen formation was observed in the negative controls, including PDMS–cGQD in the dark and PDMS under both light and dark conditions (Supplementary Figure S1).

### 3.6. The Fungicidal Activity of cGQD Thin Film on PDMS

The surface coating of cGQDs applied on PDMS (Figure 4) was tested for fungicidal activity using the JIS assay. After 30 min of illumination, at least 99.9% of the inoculum was killed for both *C. auris* and *C. albicans* (Figure 5a,b). The PDMS non-coated control and the coated PDMS dark condition showed no significant decrease in CFUs for either species. Taken together, these data show potent surface fungicidal activity of cGQDs coated on PDMS, one of the major polymers used for manufacturing catheters.

### 3.7. cGQD Thin Film on a Peritoneal/Cardiac Catheter

**Figure 6:**
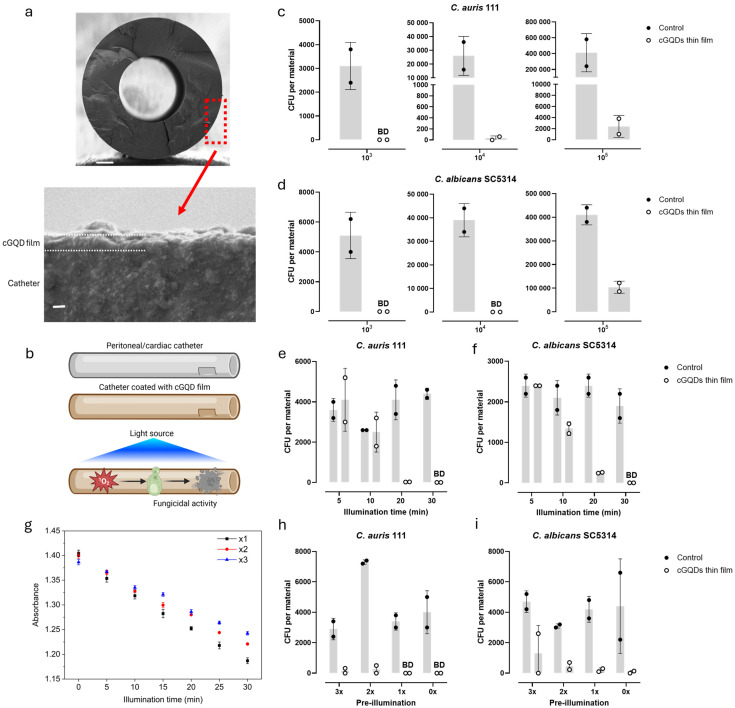
Catheter–cGQD thin films. (**a**) Cross-sectional SEM images of a catheter coated with cGQDs. Top: low-magnification view of the entire catheter cross-section (scale bar: 300 μm). Bottom: high-magnification image of the coated catheter surface showing the cGQD thin film (scale bar: 200 nm). (**b**) Schematic illustration of the antifungal mechanism of the catheter–cGQD thin film. Fungicidal activity of catheter–cGQD thin films with 30 min of blue-light illumination against different inocula of (**c**) *C. auris* and (**d**) *C. albicans*. Illumination time-series of catheter–cGQD thin film against (**e**) *C. auris* and (**f**) *C. albicans*. (**g**) ABDA absorbance decay at 380 nm during blue-light illumination over three consecutive activation cycles (x1–x3). Repeated 20 min pre-illumination of catheter–cGQD thin film against (**h**) *C. auris* and (**i**) *C. albicans*. Bars represent mean values of one experiment with duplicate measurements. Survival of yeasts is shown in CFUs per material. Detection limit is 10 CFUs.

To assess applicability of the cGQD thin film on a catheter surface, we applied our coating procedure to peritoneal/cardiac catheters. The catheters were coated with a ~205 nm thick cGQD film using the same layer-by-layer procedure applied for glass and PDMS surfaces (Figure 6a). Upon blue-light illumination, the cGQD-coated catheter generated singlet oxygen in the surrounding medium, resulting in the killing of the fungi (Figure 6b). We assessed the fungicidal capacity of the cGQD-coated catheter by adding different numbers of cells in three droplets of 1 μL per catheter segment. For *C. auris*, an inoculum of 10^3^ CFUs was reduced to below the detection limit and inocula of 10^4^ and 10^5^ CFUs were reduced by at least 99% (Figure 6c). *C. albicans* was reduced below the detection limit for inocula of 10^3^ and 10^4^ CFUs, and a 75% reduction in CFUs was reached for an inoculum of 10^5^ CFUs (Figure 6d).

To study the minimal necessary illumination time for cGQD thin film catheters, we performed the JIS assay with illumination times of 5, 10, 20 and 30 min. After 10 min, a reduction in CFUs for *C. albicans*, but not for *C. auris*, was observed. After 20 min, the entire 10^4^ CFU inoculum of *C. auris* and 90% of the inoculum of *C. albicans* was killed (Figure 6e,f). After 30 min, the entire inoculum of both *C. auris* and *C. albicans* was killed. To examine the feasibility of multiple activation cycles for the cGQD thin film-coated catheters, we monitored singlet oxygen generation over three consecutive illumination rounds, each performed in the presence of fresh ABDA probe solution. The cGQD thin film catheters generated singlet oxygen reproducibly in all three rounds, with only a slight decrease in the production level during the second and third cycles (Figure 6g). Subsequently, based on the fungicidal activity of the cGQD thin film at 20 min of illumination, we performed a repeated illumination JIS assay to assess antifungal capacity after multiple rounds of illumination. Materials were exposed to 20 min of illumination without the addition of inoculum (pre-illumination) to test a possible reduction in ROS-generating capacity after repeated illumination. Instead of inoculum, three droplets of 1 μL of demi water were added to all materials to allow ROS production upon illumination. After one, two, or three rounds of pre-illumination, the inoculum of 1 × 10^4^ CFUs was added in three droplets of 1 μL before a final round of illumination. For *C. auris*, even three pre-illuminations of 20 min did not affect the killing capacity of the cGQD thin film (Figure 6h). *C. albicans* killing was affected after three pre-illuminations, but still at least half of the inoculum was killed (Figure 6i). Taken together, we conclude that the cGQD thin film applied on a catheter has strong fungicidal activity against both *C. auris* and *C. albicans* after only 20 min of illumination, with the possibility of illuminating multiple times and maintaining capacity to eradicate yeast cells.

## 4. Discussion

Catheter-related blood stream infections caused by *Candidozyma* and *Candida* species occur most frequently in immunocompromised patients and are difficult to treat due to increasing resistance to antifungals and the formation of biofilms [6,14,16,26]. In particular, the globally emerging *C. auris* has caused large nosocomial outbreaks and infections are increasing at an alarming rate [27]. Particularly, insertion sites of indwelling devices, such as catheters, can serve as a point of entry for pathogens [28]. In this study, we show the potential of novel photosensitizers of cGQDs for the prevention of *C. auris* and *C. albicans* infections, and the feasibility of incorporating these cGQDs into a thin film for application on medical devices such as catheters.

In order to assess the fungicidal activity of our cGQDs, we analyzed their potential to kill very-difficult-to-treat *C. auris* strains. The strains we analyzed, a panel spanning clades I to V, showed expected levels of susceptibility to conventional antifungals as reported for these clades [12,13,14,15]. Interestingly, there was no correlation between the antifungal resistance profile and susceptibility to cGQDs, as all strains, even the most resistant ones, were rapidly killed by the cGQDs. The colloidal cGQDs showed 99.9% killing of planktonic yeast at a concentration of 12.5–25 μg/mL for all *C. auris* strains and 25 μg/mL for the *C. albicans* strain.

In our previous study, we investigated the cGQDs for bactericidal activity against *Staphylococcus aureus* and *Escherichia coli* [21]. Our carboxyl-functionalized GQDs generate an exceptionally high yield of singlet oxygen due to enhanced spin–orbit coupling between singlet and triplet states compared to unfunctionalized GQDs [21]. Singlet oxygen is produced via the type II reactive oxygen species (ROS) pathway, where energy is transferred to nearby ground-state (triplet-state) oxygen to produce an excited state (singlet oxygen) [29]. In general, singlet oxygen primarily kills by causing oxidative damage to cellular components, such as proteins, lipids and DNA, resulting in the destruction of cells [30,31,32]. Our results show a difference in susceptibility to colloidal cGQDs between fungal species, where the *C. auris* strains were slightly more susceptible than the *C. albicans* strain. Although only one *C. albicans* strain was tested, this result may suggest a difference between *C. auris* and *C. albicans* in catalase activity or other enzymes that neutralize ROS, or a difference in surface proteins that may not be sensitive to oxidation by singlet oxygen. However, to the best of our knowledge, no resistance evolution to aPDT for fungi or bacteria has been reported, although intrinsic differences in susceptibility between species and even strains may exist [33,34].

To assess the capacity of cGQDs to prevent survival on surfaces of different composition, we studied their potential when applied as a thin film on glass and PDMS. On both surfaces the thin film effectively killed both *C. auris* and *C. albicans*. This high efficacy is likely due to high local density of the cGQDs and the short distance to yeast cells. Singlet oxygen is very reactive, but has a short half-life of 10^−5^ s and therefore benefits from close proximity to the target [35]. As we did not observe any release of cGQDs from the thin film, most of its fungicidal effect must have come from this close contact of the cGQD thin film, the generated singlet oxygen and the fungal cells. The strong fungicidal activity and the absence of detectable leaching of GQDs make the thin film a stable coating as required for medical applications like catheters.

*C. albicans* and *C. auris* are amongst the most common pathogens causing catheter-related blood stream infections [4]. The reported (experimental) antimicrobial coatings on catheters are mainly based on traditional antifungals [36,37] or nanoparticles such as silver or gold [38]. Antifungal coatings can be effective, but their effectiveness is highly influenced by the rising antifungal resistance, especially in *C. auris*. Silver or gold catheter coatings show promising fungicidal activity but often have high toxicity for mammalian cells which makes clinical application difficult. An advantage of the use of cGQDs is their low cytotoxicity both in colloidal form and incorporated into a thin film [21]. Recently, Gupta et al. developed a chlorhexidine and chlorhexidine–silver sulfadiazine-impregnated central venous catheter which was effective in vitro against *C. auris* [39]. However, the study reported that the coating may not be suitable for long-dwelling catheters and no cytotoxicity studies were performed. In another study, Chen et al. reported a stable antibacterial coating based on aggregation-induced emission luminogens photosensitizers on glass and PDMS, which had strong antibacterial activity, but was not tested for antifungal activity [40]. Similarly, most applications specifically based on graphene quantum dots have been evaluated primarily against bacteria, but not fungi [41,42,43,44]. To the best of our knowledge, there are no other catheter coatings based on GQDs which have been tested against *C. auris* or *C. albicans*. Therefore, the application of cGQD thin films on catheters points out a promising solution.

The entry point for catheter-related infection is often considered to be the site of catheter insertion into the skin [28]. This site can be reached with external illumination and, for our cGQD coating, can be illuminated repeatedly with sustained fungicidal activity. To reach intracorporal parts of the catheter, cGQD-coated catheters with incorporated optical fibers could be developed [45,46]. This way, colonization and biofilm formation on the indwelling parts of catheters could be prevented, or existing biofilms even eradicated.

In summary, cGQDs, both in suspension and when integrated onto flexible medical catheter surfaces, exhibited potent fungicidal activity against the resilient pathogens *C. auris* and *C. albicans*. These nanomaterials represent a significant alternative toward preventing healthcare-associated infections caused by multi-drug-resistant fungi.

## Figures and Tables

**Table 1:** Susceptibility of *C. auris* strains for conventional antifungals based on minimum inhibitory concentration (MIC) determined by broth microdilution according to EUCAST guidelines. Resistance cut-offs as determined by EUCAST are >2 μg/mL for amphotericin B and >0.25 μg/mL for micafungin [25]. Breakpoints for other antifungals have not yet been defined for *C. auris*. Values are in μg/mL.

Strain Information		Triazole Class					Polyene Class	Echinocandin Class
Strain	Clade	Fluconazole	Voriconazole	Itraconazole	Isavuconazole	Posaconazole	Amphotericin B	Micafungin
111	I	>64	2	0.25	0.25	0.063	1	4
2MG-A0203-99	I	0.5	<0.008	<0.008	<0.008	<0.008	1	0.5
2MG-A0203-100	I	>64	1	0.5	0.25	0.125	4	0.25
2MG-A0102-44	II	2	0.015	0.015	<0.008	<0.008	1	0.031
2MG-A0102-51	II	64	0.125	0.25	0.25	0.031	1	0.125
2MG-A0202-80	III	>64	4	0.5	0.25	0.063	1	>4
2MG-A0203-98	III	>64	1	0.125	0.125	0.125	1	>4
2MG-A0203-50	IV	64	2	0.5	1	0.25	1	0.5
2MG-A0203-51	IV	>64	0.5	0.5	0.5	0.125	2	0.5
2MG-A1002-43	V	>64	2	2	2	2	2	2
2MG-A1703-88	V	>64	0.25	0.25	0.25	0.25	1	0.25

**Table 2:** Minimum fungicidal concentration (MFC) of cGQDs against *C. auris* isolates of different clades determined by MFC assay.

Strain	Clade	Minimum Fungicidal Concentration of Colloidal cGQD (μg/mL)
111	I	12.5–25
2MG-A0203-99	I	12.5
2MG-A0203-100	I	12.5
2MG-A0102-44	II	12.5
2MG-A0102-51	II	12.5
2MG-A0202-80	III	25
2MG-A0203-98	III	25
2MG-A0203-50	IV	12.5
2MG-A0203-51	IV	12.5
2MG-A1002-43	V	12.5
2MG-A1703-88	V	12.5

## Data Availability

The original contributions presented in this study are included in the article.

## Supplementary Materials

The following supporting information can be downloaded at: https://www.mdpi.com/article/10.3390/jof12030216/s1, Figure S1: (a, b) Absorption spectra of the ABDA singlet oxygen probe measured over 30 min for PDMS control and PDMS–cGQD films under dark conditions. (c) ABDA absorption under blue-light illumination in the presence of a PDMS control, demonstrating negligible singlet oxygen generation from the polymer matrix.
