# Supplementary material for: A Carboxyl-Functionalized Graphene Quantum Dot Coating for Catheters Effective Against Emerging Drug-Resistant Candidozyma auris
Source: J Fungi (Basel). 2026 Mar 17;12(3):216. doi: 10.3390/jof12030216 (PMC13027909; doi:10.3390/jof12030216)
Supplement: Supplementary file 1 [file jof-12-00216-s001.zip › jof-4186510-supplementary.pdf]

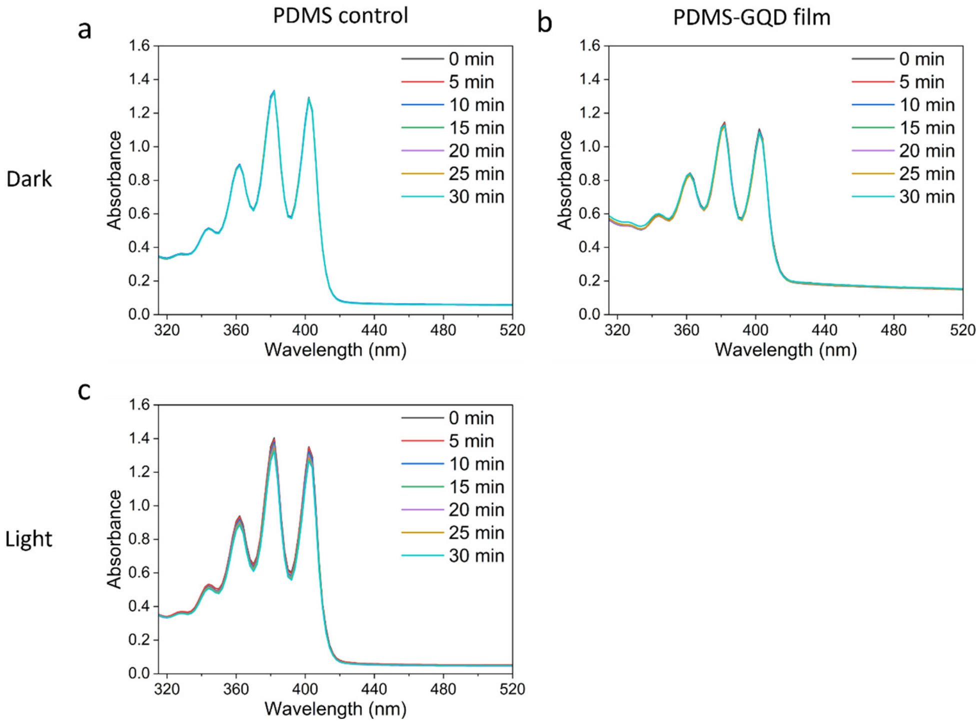

Figure S1. (**a**,**b**) Absorption spectra of the ABDA singlet oxygen probe measured over 30 min for PDMS control and PDMS–cGQD films under dark conditions. (**c**) ABDA absorption under blue-light illumination in the presence of a PDMS control, demonstrating negligible singlet oxygen generation from the polymer matrix.
